# Electron heating in rf capacitive discharges at atmospheric-to-subatmospheric pressures

**DOI:** 10.1038/s41598-018-27945-6

**Published:** 2018-07-05

**Authors:** Sanghoo Park, Wonho Choe, Holak Kim

**Affiliations:** 1 Department of Nuclear and Quantum Engineering, Korea Advanced Institute of Science and Technology, 291 Daehak-ro, Yuseong-gu, Daejeon, 34141 Republic of Korea; 2 Department of Physics, Korea Advanced Institute of Science and Technology, 291 Daehak-ro, Yuseong-gu, Daejeon, 34141 Republic of Korea

## Abstract

Electron heating is a fundamental and multidisciplinary phenomenon in partially ionized gases, from the planet’s ionosphere to laboratory-scale plasmas. Plasmas produced at ambient or reduced pressures have recently shown potential for scientific and industrial applications. However, electron heating, which is strongly coupled to the physicochemical properties of these plasmas, has been poorly understood. We experimentally found the rapid structural transition from non-local to local electron heating in collisional radio-frequency discharges at atmospheric-to-subatmospheric pressures. As the gas pressure decreased from 760 to 200 Torr, the time-averaged electron density increased from 1.3 × 10^12^ to 1.3 × 10^13^ cm^−3^, and the electron temperature decreased from 2.5 to 1.1 eV at the maximum allowable discharge current in the abnormal α-mode in the plasma bulk. The spatiotemporal evolution of the electron temperature clearly shows that the electron temperature increases uniformly throughout the bulk plasma region during sheath expansion and collapse at 760 Torr, but the electron heating weakens with sheath collapse as the gas pressure decreases.

## Introduction

Most natural phenomena, to our knowledge, are associated with gas pressure and arise from pressure changes. For ionized gases, including those in the earth’s atmosphere and laboratory-scale plasmas, it is also one of the most crucial parameters that influence the ionization process because it is related to electron-neutral collisional coupling and electron mobility. In the earth’s ionosphere, where the gas pressure is under 10^−6^ bar, free electrons and ions readily respond to the electromagnetic field and affect geophysical phenomena, which do not appear in the lower atmosphere^1–3^. Advances in knowledge through scientific studies on electron kinetics and heating at given pressure have unveiled anomalous phenomena and their mechanisms. Alongside with ionized gases in the atmosphere, many studies have investigated the pressure dependence of the electron characteristics of laboratory plasmas, and previous works have shown the great influence of gas pressure on plasma properties^4,5^. However, in contrast to low-pressure plasmas (e.g., under 1 mTorr), plasma properties in the high-pressure regime, including atmospheric pressure, are not well understood; yet, the electron diagnostic for such plasmas still remains a significant challenge. Due to the different plasma characteristics at different pressures in the subatmospheric-to-atmospheric pressure range, characterizing the plasma over such a pressure range is a prerequisite for understanding the underlying principles of plasmas and for industrial plasma applications^6^. One example is the dielectric barrier discharge (DBD). Because of the potential implementation of DBD actuators as aerodynamic devices in gas turbines and airplanes, the effects of gas pressure on the characteristics of DBD actuators have been actively investigated over the expected range of pressures^7,8^.

Over the past several decades, plasmas generated at ambient pressure have received great attention as a multidisciplinary topic due to their complexity, and there has been a dramatic increase in plasma applications. Recent publications clearly demonstrate possible utilizations of atmospheric-pressure plasmas and their outstanding results^9–12^. In most applications, various plasma sources are used as generators of reactive chemical species because the contribution of plasmas to processing targets is dominated by chemical reactions. Moreover, short-lived reactive species, which are key players in most plasma applications, are remotely produced and controlled via the photolysis or post-discharge reactions of long-lived species^13–16^. Thus, direct contact of the target with the plasma or releasing the plasma into ambient air is not a key requirement in certain applications, and attempts to utilize plasmas in the subatmospheric pressure range have recently increased. However, as previously mentioned, despite the remarkable demand and interest in plasmas at atmospheric-to-subatmospheric pressures, not many studies have been performed to determine the effect of gas pressure on the electron kinetics, which are directly coupled to the chemical reactions in plasmas near atmospheric pressure. This is mainly because suitable and available electron diagnostics for these plasmas are lacking.

Here, we report the electron properties, i.e., electron density (*n*_e_) and temperature (*T*_e_), and electron kinetics of radio-frequency (rf) argon capacitive discharges in a pressure range from 200 to 760 Torr. To understand the electron properties under different pressure condition, we have experimentally investigated the electron heating structures during an rf cycle based on the measured spatiotemporal distribution of the neutral bremsstrahlung and electron temperature. The results clearly demonstrate the rapid structural transition from non-local to local electron heating; the electron heating during the sheath collapse weakens as the pressure decreases.

## Results

**Figure 1:**
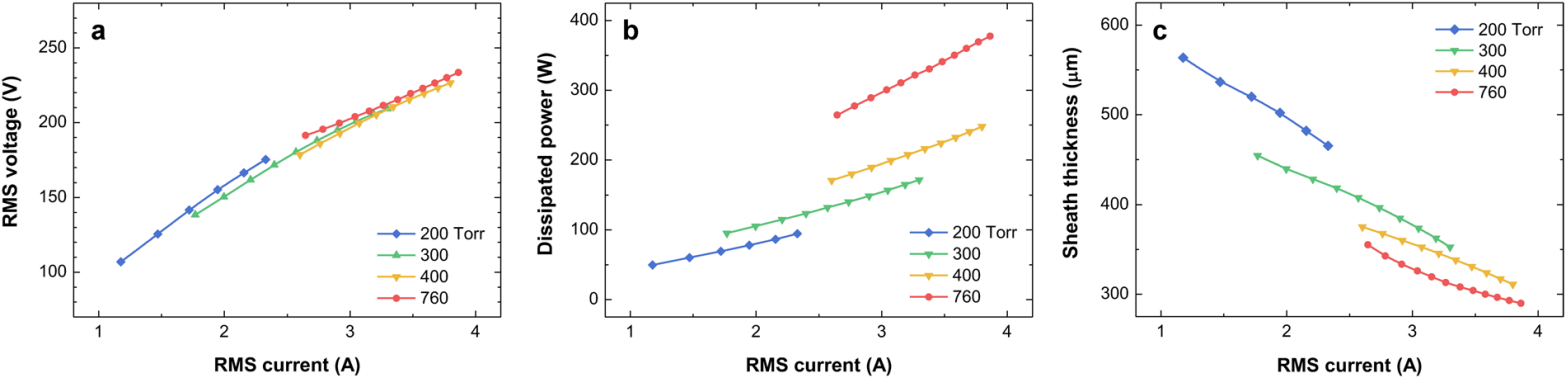
Electrical characteristics of argon rf discharge at 200–760 Torr. (**a**) *I*_rms_-*V*_rms_ and (**b**) *I*_rms_-*P*_dis_ characteristics; (**c**) the sheath thickness of argon capacitive discharges at various pressures. The leftmost and rightmost data points of the 200, 300, 400 Torr discharges indicate the minimum and maximum attainable conditions, respectively, in the abnormal α-mode, whereas the maximum operation condition of the α-mode for the 760 Torr discharge are not presented due to the limited power supply capacity.

The root mean square (rms) current versus the rms voltage (*I*_rms_-*V*_rms_) and the rms current versus the power dissipated in the plasma (*I*_rms_-*P*_dis_) curves are shown in Fig. 1(a,b), respectively. As all the discharges were operated in the abnormal α-mode, *V*_rms_ and *P*_dis_ are almost linearly proportional to *I*_rms_. As presented in the figure, the accessible ranges of *V*_rms_ and *P*_dis_ decrease with the decreasing pressure. The slopes of the *I*_rms_-*V*_rms_ curves slightly increase with the decreasing pressure. According to the one-dimensional (1-D), simple resistor-capacitor series circuit model, which is an acceptable model for atmospheric-pressure capacitive discharges^6^, the slope of the *I*_rms_-*V*_rms_ curve is roughly $1/2{\rm{\pi }}(13.56[{\rm{MHz}}])/(1.52{\varepsilon }_{0}S/d)$, where *ε*_0_ is the permittivity in a vacuum, *S* is the cross sectional area of the plasma, and *d* is the sheath thickness. Therefore, an increase in the slope as the pressure decreases indicates an increase in the sheath thickness. Consequently, because the voltage drop across the sheath increases as the sheath thickness increases at the same discharge current, the rf power that dissipates in the plasma decreases as the gas pressure decreases, as seen in Fig. 1(b). An analytical solution for a collisional sheath^17^ presents the relation between the gas pressure, *p*, the sheath thickness, *s*_m_. The thickness of an ion-dominated collisional sheath is expressed as

1 $${s}_{{\rm{m}}}=1.95{(\frac{2{\delta }_{{\rm{i}}}}{{{\rm{\pi }}}^{2}{\delta }_{{\rm{D}}}})}^{1/2}{\{\frac{J}{e(2{\rm{\pi }}f){n}_{0}}\}}^{3/2},$$

where ${\delta }_{{\rm{i}}}[{\rm{cm}}]={(300\times p[{\rm{Torr}}])}^{-1}$ is the ion mean free path for an argon discharge, *δ*_D_ is the electron Debye length, *J* is the rf current density passing through the sheath, *e* is the elementary charge, *f* is the driving frequency, *n*_0_ is the electron density at the sheath edge. This solution indicates that the sheath thickness is proportional to *p*^−1/2^. The total sheath thickness, which was evaluated by the circuit model using the phase difference between the voltage and current and other electrical factors, is plotted as a function of *I*_rms_ in Fig. 1(c). The maximum sheath thickness of 564 μm at 200 Torr is approximately two times larger than the minimum sheath thickness of 290 μm at 760 Torr. As intuitively and analytically expected, the sheath thickness of an argon capacitive discharge increases with the decreasing pressure at a constant discharge current.

**Figure 2:**
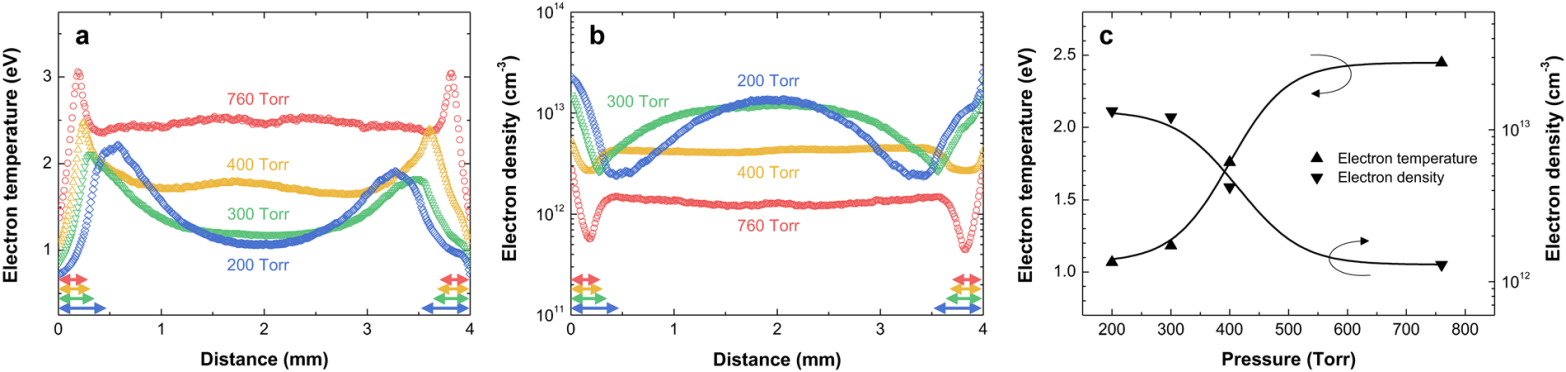
Dependence of the electron characteristics on the gas pressure. Profiles of time-averaged (**a**) *T*_e_ and (**b**) *n*_e_ along the axis perpendicular to the electrodes; the (**c**) electron density and temperature at the center of the gas gap in the profiles as a function of gas pressure. All cases at each pressure correspond to the rightmost data of the characteristic curves in Fig. 1. The colored arrows in (**a**,**b**) indicate the sheath thickness given in Fig. 1(c).

In the following, the relationship between the gas pressure and electron properties at atmospheric-to-subatmospheric pressures is discussed. First, the cut-away views of the time-averaged *T*_e_ and *n*_e_ profiles relative to the electrode are presented in Fig. 2(a,b), respectively (two-dimensional distribution of neutral bremsstrahlung and *T*_e_ can be found as Supplementary Fig. S1). The measured *T*_e_ profiles near each electrode are partially inconsistent with the numerical modelling results^18,19^. The profiles presented in Fig. 2(a) show that the maximum *T*_e_ exists slightly away from the electrodes, and *T*_e_ decreases towards both the electrodes and bulk. In comparison, the simulation results show that the *T*_e_ in the plasma sheath (from the electrode surface to the plasma-sheath boundary; the location of the highest *T*_e_ is considered to be the plasma-sheath boundary in this paper) is the highest and generally higher than 4 eV. This discrepancy in the sheath region may be attributed to the non-Maxwellian nature of the electron energy distribution, particularly near the electrodes. The uncertainty in the electron diagnostics used in this work increases when the electron energy distribution function distorts from a Maxwellian distribution because the neutral bremsstrahlung emissivity was calculated based on a Maxwellian electron energy distribution. In the plasma sheath, a non-Maxwellian distribution can be caused by cold electrons; these are produced within the sheath region through the ionization processes initiated by the secondary electrons emitted from the metallic electrode or dielectric surfaces. Thus, the estimated *n*_e_ and *T*_e_ in the vicinity of the electrodes [see Fig. 2(a,b)] may possibly deviate from the real values.

At atmospheric pressure, the *n*_e_ and *T*_e_ are more or less constant throughout the bulk plasma, and the values are approximately 10^12^ cm^−3^ and 2.5 eV, respectively, except near the electrodes. The rapid change in the *n*_e_ and *T*_e_ near the electrode is due to the frequent collisions between the electrons and neutrals that hinder the energetic electrons from moving the electrode gap distance. As the pressure decreases, the mean free path of the electron increases, resulting in a smooth profile over the entire gas gap. As discussed in the foregoing paragraph, the distance of the highest *T*_e_ from the electrode, which corresponds to the sheath width, increases with decreasing pressure [see Fig. 1(c)]. A noticeable difference between the profiles of *n*_e_ and *T*_e_ is observed in Fig. 2(a,b) as the gas pressure changes, and the difference is presented in Fig. 2(c). The *n*_e_ and *T*_e_ plotted in the figure represent the values at the center of the gas gap for each pressure. As depicted in the figure, *T*_e_ decreases (from 2.5 to 1.1 eV) while *n*_e_ increases (from 1.3 × 10^12^ to 1.3 × 10^13^ cm^−3^) with decreasing pressure. This relation of *n*_e_ and *T*_e_ with the pressure can be intuitively interpreted as follows. As the gas pressure decreases, the electrons and metastable Ar atoms that are produced at the plasma-sheath boundary can diffuse quickly enough to overcome the rf oscillating field due to the increased mean free path, thereby they move further towards the electrode and plasma bulk region. Additionally, near atmospheric pressure, the dominant electron heating mechanism is ohmic (collisional) heating due to frequent particle collisions. Thus, the electron heating is strongly governed by the electric field; i.e., the *n*_e_ and *T*_e_ distributions are affected by the electric field distribution and vice versa. In other words, the electron production in the field-enhanced region, which is built up by space charges in the plasma-sheath boundary, weakens with decreasing pressure during the sheath collapse. As a result, the *n*_e_ profile becomes a convex profile with a maximum in the plasma bulk as the pressure decreases instead of a weakly concave profile with two maxima near the electrodes. By considering the power balance, the *T*_e_ profiles can be estimated from the *n*_e_ profiles. The number density of the electrons increases with decreasing pressure in the plasma bulk, and consequently, the power absorbed per electron decreases in the plasma bulk.

**Figure 3:**
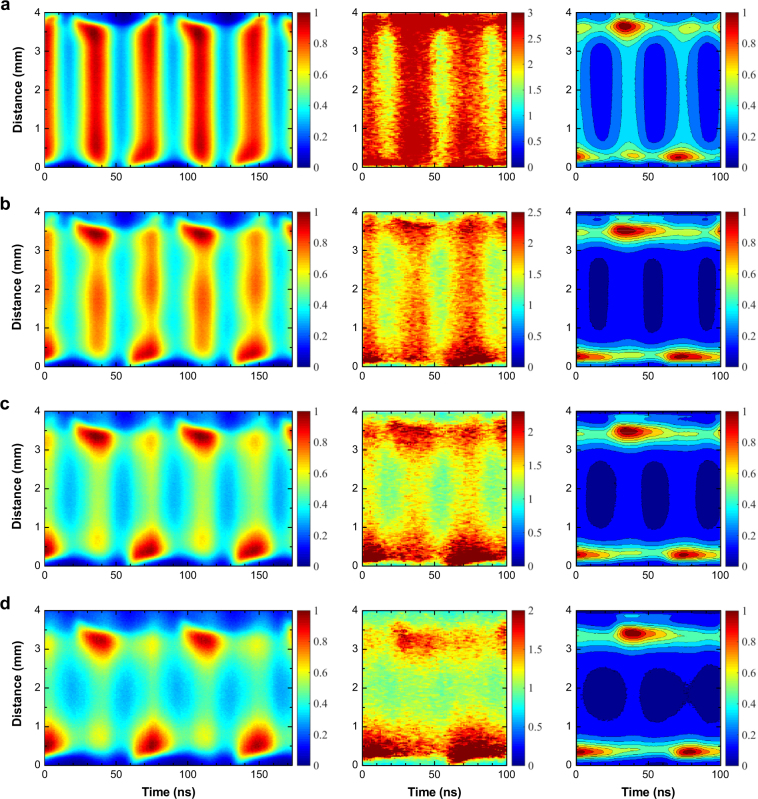
Nanosecond-resolved visualization of the electron heating structure. Spatiotemporal evolution of 514.5-nm continuum radiation (1st column), *T*_e_ (2nd column), Ar I emission (3rd column) at (**a**) 760 Torr, (**b**) 400 Torr, (**c**) 300 Torr, (**d**) 200 Torr. The intensities of the continuum radiation and Ar I emission are normalized by the maximum intensity, and the unit of *T*_e_ is eV. Color bars are located on the right side of each image.

For further insight into the electron kinetics and heating structures in the rf oscillating field, the space- and phase-resolved profiles of the *T*_e_ and Ar I atomic line (2p → 1s, 696.5 nm and 706.7 nm) emission were obtained using an intensified charge-coupled device (iCCD) camera with ultra-fast gating (see Materials and Methods Section and Supplementary Materials for details on the imaging technique for electron heating structure). The spatiotemporal evolution of the continuum radiation, *T*_e_, the Ar I line emission in argon capacitive discharges operated at 200, 300, 400, 760 Torr are shown in each column of Fig. 3.

The neutral bremsstrahlung images demonstrate the periodic behaviour of the plasma-sheath boundary and the electron heating structure for all the gas pressures, and the *T*_e_ and Ar I emission profiles explicitly show the pressure dependence of the spatiotemporal behaviour of the electrons. As shown in Fig. 3(a), the 514.5-nm continuum emission and *T*_e_ increase during the sheath expansion and retreat phases at 760 Torr, and their profiles have symmetric and non-local structures with respect to the center of the gas gap, which indicates simultaneous electron heating near both electrodes. A similar distribution is found in the spatiotemporal evolution of the Ar I emission, which is shown in the rightmost image in Fig. 3(a). Under high-pressure conditions, electron heating is due to the formation of a field-enhanced region caused by space electrons and ions at the retreating sheath edge. Due to the high collision rate, the electron motion is limited, and as a result, the localized electric field induced by the space electrons accelerates the electrons towards the electrode during the sheath collapse. Although heating is negligible at the edge of the retreating sheath in low-pressure discharges^20^, a similar heating mechanism was observed when electrons were subjected to increased collisions in the presence of molecular gases^21^. However, electron heating is accompanied by a field reversal during the retreating sheath in low-pressure discharges^21^, whereas there is no field reversal in the retreating sheath of rf capacitive discharge at atmospheric pressure^22^. As shown in Fig. 3 and detailed in Supplementary Fig. S2, the emission intensity and *T*_e_ during the sheath collapse become lower than those during the sheath expansion as the pressure decreased because charged species, including electrons, can diffuse sufficiently fast during the half rf period. A numerical simulation solving the 1-D fluid equations^22^ showed that the heating in the neighbourhood of the retreating sheath decreases rapidly with decreasing pressure, which is quite consistent with our observation.

One noticeable feature is the pressure dependence of the electron heating profile in the direction perpendicular to the electrodes. As noticed in the time-averaged *T*_e_ distribution [see Supplementary Fig. S1(e–h) and Fig. 2(a)], the electron temperature in the plasma bulk region decreases with decreasing pressure, resulting in a crater-like profile shape. As discussed above, the ohmic heating caused by the field-enhanced region built up by the space charge is depressed with decreasing pressure, resulting in the *T*_e_ decreasing.

As shown in the leftmost column of Fig. 3, the width of the weak-intensity area corresponding to the electron-depleted regions near the electrodes (sheath edges) increases with decreasing pressure. This result is consistent with the relation between the sheath thickness and pressure obtained from the time-averaged *T*_e_ profiles and *I*_rms_-*V*_rms_ characteristics, as seen in Supplementary Fig. S1and Fig. 1(c).

## Discussion

Our findings noted that the pressure change from atmospheric to subatmospheric pressures results in a rapid transition of electron heating in partially ionized gases. The model experiment was based on capacitively coupled argon plasma at 200–760 Torr. The sheath thickness, which was estimated by both the electric circuit model and experimental *T*_e_ images, shows an increasing trend with decreasing pressure. As the gas pressure decreased, the time-averaged *n*_e_ increased from 1.3 × 10^12^ to 1.3 × 10^13^ cm^−3^ at the maximum allowable discharge current in the abnormal α-mode, while *T*_e_ decreased from 2.5 to 1.1 eV. We have clearly demonstrated that the electron heating structures of discharges are significantly different in the pressure range from 200 to 760 Torr. The electron temperature increases uniformly throughout the plasma bulk region during the sheath expansion and collapse at 760 Torr. However, the spatiotemporal evolution of the continuum radiation (neutral bremsstrahlung) and *T*_e_ indicate that the local electron heating during the sheath collapse, which is ohmic heating caused by space charges, weakens as the gas pressure decreases. Even at this very moment electron heating occurs continuously in ionized gases and impacts on natural phenomena. The results that can serve as a basic and informative reference for future scientific research is of paramount importance for scientific impact. Moreover, this report provides the fundamental knowledge of the electron heating in partially ionized gases in any plasma, ranging from plasma processing, astrophysics to space propulsion as well.

## Materials and Methods

### Information about the plasma apparatus

**Figure 4:**
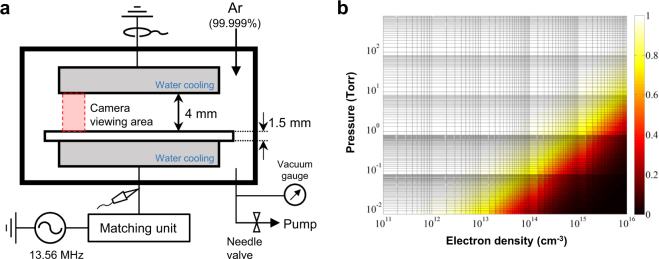
Plasma apparatus and allowable plasma characteristics for neutral bremsstrahlung-based electron diagnostics. (**a**) Schematic of a plasma chamber and relevant experimental setup for producing a parallel-plate capacitive discharge in the pressure range 200–760 Torr. (**b**) Fraction of the neutral bremsstrahlung emissivity, ${\kappa }_{{\rm{ea}}}/({\kappa }_{{\rm{ea}}}+{\kappa }_{{\rm{ei}}}^{{\rm{ff}}}+{\kappa }_{{\rm{ei}}}^{{\rm{fb}}})$, with 3.0-eV *T*_e_ at 300 nm as a function of the gas pressure and electron density. Neutral bremsstrahlung-based electron diagnostics is valid under the conditions in the white region.

The present study was performed using a plasma chamber designed to facilitate optical diagnostics and plasma generation in the pressure range from 200 to 760 Torr. A schematic illustration of the capacitive plasma source and relevant system is shown in Fig. 4(a). Two rectangular electrodes with a plasma-facing area of 138 × 60 mm^2^ were parallelly installed inside the chamber and cooled by city water to maintain the electrode temperature at 25°C. A sinusoidal 13.56-MHz rf power supply (RFPP RF10S) was connected to the bottom electrode through an impedance matching circuit, and the upper electrode was grounded. A 1.5-mm thick alumina plate was used to cover the entire surface area of the powered electrode as a dielectric barrier. The gap distance between the bare upper electrode and the alumina plate was fixed at 4 mm for all experiments. To obtain the electrical characteristics of the discharges, a wide-band voltage probe (Tektronix P6015A) and a current probe (Tektronix TCP202) were used with an oscilloscope (Tektronix TDS3012B). The following procedure was used to produce plasma. After pumping to near 20 mTorr using a rotary pump, 99.999% purity argon gas was supplied at 1.2 slpm (standard liters per minute) into the chamber through a mass flow controller (MKS 1179A). Likewise, the argon purge was performed several times before every experiment; it was repeated until the electric characteristics of plasma reach steady state. Subsequently, the argon gas was continuously supplied into the chamber throughout experiment at 1.2 slpm, and the gas pressure was monitored by a vacuum gauge (MKS 626A) and controlled by adjusting a needle valve installed at the pump inlet. Throughout the experiment, the gas supply and pumping systems were continuously operated to maintain the gas pressure at a specific level.

### Electron diagnostics based on electron-neutral bremsstrahlung

Electron diagnostics, which is based on neutral bremsstrahlung, was used in this work. Continuum radiation emitted from weakly ionized gases mainly originates from electron-neutral interactions, i.e., neutral bremsstrahlung, and its emissivity (*κ*_ea_) contains electron information^23–26^. Because the contributions of other continuum radiation sources, electron-ion (free-free) bremsstrahlung $({\kappa }_{{\rm{ei}}}^{{\rm{ff}}})$ and (free-bound) recombination $({\kappa }_{{\rm{ei}}}^{{\rm{fb}}})$, to the emissivity in the UV and visible range vary with the plasma driving conditions, particularly the gas pressure, *κ*_ea_ dominant conditions should be assured. A simple calculation using equations (1–3), which were described in the previous paper^23^, with *T*_e_ = 3 eV, *n*_e_ = *n*_i_, and wavelength dependent Biberman factors indicates that ${\kappa }_{{\rm{ea}}}\gg {\kappa }_{{\rm{ei}}}^{{\rm{ff}}}+{\kappa }_{{\rm{ei}}}^{{\rm{fb}}}$ at 300 nm when *n*_e_/*n*_a_ < 10^−3^ [*n*_i_ and *n*_a_ are the ion and neutral (gas) density], which is the case for most low-temperature plasmas at subatmospheric-to-atmospheric pressure. A *κ*_ea_ dominant range, i.e., ${\kappa }_{{\rm{ea}}}/({\kappa }_{{\rm{ea}}}+{\kappa }_{{\rm{ei}}}^{{\rm{ff}}}+{\kappa }_{{\rm{ei}}}^{{\rm{fb}}}) \sim \,1$, corresponding to valid plasma conditions for neutral bremsstrahlung-based diagnostics, can be seen in the color scale ${\kappa }_{{\rm{ea}}}/({\kappa }_{{\rm{ea}}}+{\kappa }_{{\rm{ei}}}^{{\rm{ff}}}+{\kappa }_{{\rm{ei}}}^{{\rm{fb}}})$ in Fig. 4(b). The *n*_e_ profile in Fig. 2(b) was estimated based on the 514.5-nm neutral bremsstrahlung and *T*_e_ profiles; the neutral bremsstrahlung emissivity can be expressed as ${\kappa }_{{\rm{ea}}}(\lambda )={n}_{{\rm{e}}}{n}_{{\rm{a}}}C\times f({T}_{{\rm{e}}})$, and the electron density is ${n}_{{\rm{e}}}={\kappa }_{{\rm{ea}}}(\lambda )/{n}_{{\rm{a}}}C/f({T}_{{\rm{e}}})$, where *C* is a constant; all the parameters in the right-hand side of the equation are known.

### Time-averaged and Time-resolved 2-D electron temperature measurement

The technique for imaging 2-D distribution of *T*_e_ is found in our previous papers^27,28^, and practical considerations of this technique are provided in Supplementary Materials. For measuring the time-averaged *T*_e_ profiles, the continuum radiation at two different wavelengths were acquired using a combination of optical interference filters having ultra-narrow transmittances with center wavelengths of 514.5 nm and 632.8 nm and an intensified charge-coupled device (iCCD) camera (Andor DH312T) with a 0.5-s exposure. Two hundred shots were averaged for a single image to reduce the instrumental noise. Using the same materials, spatiotemporally resolved *T*_e_ profiles were obtained as follows. Phase-resolved sequential images of the continuum radiation at 514.5 nm and 632.8 nm were obtained with a gate width of 6 ns on the iCCD camera, and the interval between two time-adjacent shots was 1 ns (see Fig. S4). All shots were acquired with a 2-s exposure time, and five shots were averaged to produce a single image. The 452-kHz trigger signal, which is 1/30 of 13.56 MHz, for the iCCD camera was provided using a signal generator (Agilent 33512B), and the signal was synchronized with the rf power supply. A single shot was captured using the Integrate-on-chip mode of the iCCD camera, in which charges were accumulated 9.04 × 10^5^ (452-kHz gate signal × 2-s exposure time) times on the CCD. By integrating the emission profiles along the direction parallel to the electrodes, the spatiotemporal evolutions of the continuum radiation were obtained, which are presented in the leftmost column in Fig. 3. Through the same procedure, the Ar atomic emissions, which are shown in the rightmost column in Fig. 3, were obtained using an optical interference filter with a center wavelength transmittance at 700 nm and a full width at half maximum of 25 nm.

### Data availability

The datasets generated during and/or analysed during the current study are available from the corresponding author on reasonable request.

## Electronic supplementary material

Supplementary Materials
