# Supplementary material for: Electron heating in rf capacitive discharges at atmospheric-to-subatmospheric pressures
Source: Sci Rep. 2018 Jul 5;8:10217. doi: 10.1038/s41598-018-27945-6 (PMC6033924; doi:10.1038/s41598-018-27945-6)
Supplement: Supplementary file 1 — Supplementary Materials [file 41598_2018_27945_MOESM1_ESM.pdf]

Supplementary Materials

Electron heating in rf capacitive discharges at atmospheric-to-subatmospheric pressures

Sanghoo Park[1], **Wonho Choe**[1,2,*], **and Holak Kim**[2]

[1]Department of Nuclear and Quantum Engineering, Korea Advanced Institute of Science and Technology, 291 Daehak-ro, Yuseong-gu, Daejeon 34141, Republic of Korea

[2]Department of Physics, Korea Advanced Institute of Science and Technology, 291 Daehak-ro, Yuseong-gu, Daejeon 34141, Republic of Korea

*wchoe@kaist.ac.kr

Description: Supplementary Figures, Supplementary Discussions, and Supplementary References

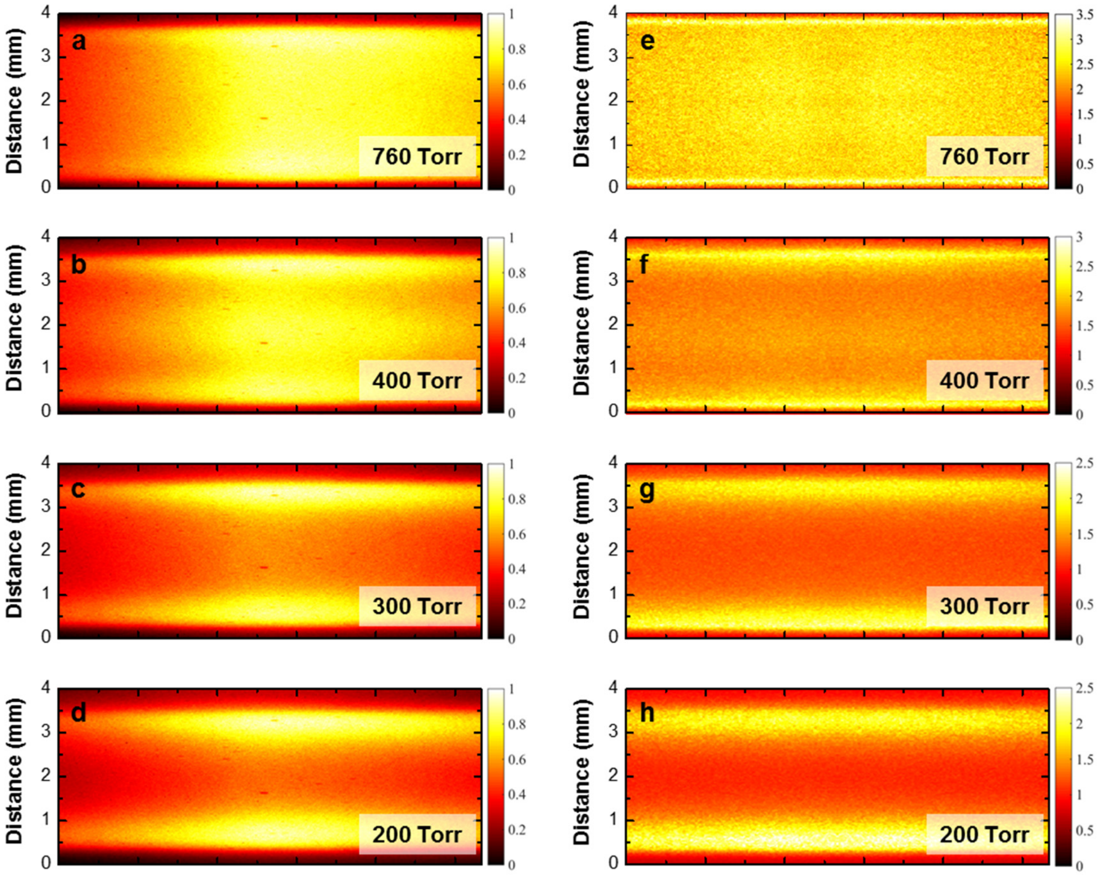

Supplementary Figure S1. Time-averaged visualization of the electron heating structure. Time-averaged two-dimensional distribution of (a-d) 514.5 nm-continuum radiation and (e-h) T_e of the argon capacitive discharges at different pressures, 760 (1st row), 400 (2nd row), 300 (3rd row), 200 Torr (4th row). All cases at each pressure correspond to the rightmost data of the characteristic curves in Fig. 1 in the main text. The same length scale is used for the image along each axis. The intensities of the continuum radiation are normalized by the maximum intensity, and the unit of T_e is eV. Color bars are located on the right side of each image.

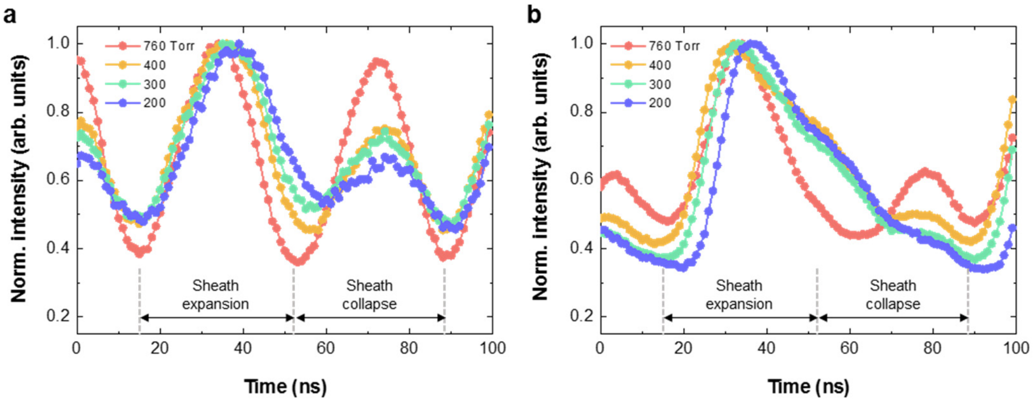

Supplementary Figure S2. Normalized intensity profiles. Temporal intensity profiles of (a) 514.5-nm continuum radiation and (b) 700-nm Ar I emission at various gas pressures of 760, 400, 300, 200 Torr extracted from Fig. 3 in the main text at certain positions near the upper electrode that exhibited a local maximum intensity. Both intensities decrease with the decreasing gas pressure during the sheath collapse phase.

Supplementary Discussions

Brief introduction of imaging techniques for electron information

According to our calculation, electron diagnostics based on neutral bremsstrahlung is available in weakly ionized gases with an electron density of less than 10^{16} cm^{-3} and an electron temperature of less than 4 eV in the pressure range from 200 to 760 Torr.

Two-dimensional (2-D) discharge images of the intrinsic optical radiation are a common and accessible way to study the spatial discharge structure.[1] In the past, an analysis of the discharge structure only involved an examination of dark and light spots in natural photographs. The basic concept underlying this approach is that when a radiation source at a given point in space is stronger, the electron density at that point is higher, and the field that imparts energy to the electrons is also higher, resulting in increasing their temperature and chance of the plasma being radiative. However, a variety of radiation sources in plasmas, including atmospheric-pressure plasmas, and imaging devices have limitations in terms of the measurable wavelength range. Thus, with this approach, identifying the major factor in the optical radiation is difficult; i.e., the spectrally unresolved discharge images cannot be accurately interpreted to understand the plasma information due to the complexity of plasma radiation. The recent trend in modern plasma diagnostics emphasizes plasma imaging and 2-D mapping of plasma parameters due to the powerful advantage of examining the inside of the plasma. A combination of imaging devices and optical filters with narrow pass bands for accurate wavelength selection has offered a cost-effective recording method for selective spectral images. Since the effective aperture of the filter is large compared to the slits, a rapid and accurate measurement is guaranteed. Because of the interference filter's unique benefits, it is widely used in various industries and in plasma studies. In the following, the instruments for 2-D imaging of T_e are based on the principle of neutral bremsstrahlung, and their issues in measurements are described. This technique is extremely simple. A 2-D distribution of T_e can be obtained from the intensity ratio of two spectrally resolved images because T_e determines the spectral distribution of the neutral bremsstrahlung emissivity.[2]

To measure the 2-D distribution of the neutral bremsstrahlung emitted at two different wavelengths, optical interference filters were used. These filters have center wavelengths at 514.5 nm and 632.8 nm and a full width at half maximum (FWHM) of transmittance (λ_{FWHM}) of < 1.5 nm. The transmittance curves of the filters are shown in Fig. S3. These two wavelengths are suitable for measuring continuum radiation in plasmas, particularly in atmospheric pressure argon capacitive discharge as discussed below. However, an error can be caused by the imperfect characteristics of the interference filter. When an interference filter is used in an optic system, it should be carefully designed due to its unique characteristics. In general, most interference filters are manufactured for incident light at a normal angle of incidence, in which the surface of the filter is perpendicular to the light path. Because the center wavelength will be shifted to a lower wavelength and the shape of the transmittance will be distorted for non-normal incident light, an accurate measurement cannot be expected. In this work, the area of interest and the size of the iris are much smaller than the lens' focal length, so the incident angle is within one degree. Therefore, the error induced by the angle of incidence was neglected. To measure the T_e of large plasmas, only the perpendicular light should be considered, and this can be accomplished using a relevant optical system, e.g., a two plano-convex lens system. The transmittance of the filters is slightly influenced by the temperature because the filters consist of dielectric layers. All the layer thicknesses increase due to thermal expansion as the temperature increases, causing the dielectric indices to change. Thus, the transmittance curve shifts slightly towards longer wavelengths. The optical density is also important in continuum radiation measurements because the radiation power of atomic lines is non-negligible at certain wavelengths. Based on the emission spectra, a sufficient optical density should be used to block undesired photons.

A more detailed procedure for the 2-D measurement of the time-averaged T_e can be found in our previous work[2]. Because the discharges oscillating with an rf power are periodically reproduced, the spatiotemporal evolution of the discharge intensity during an rf period can be recorded by the

intensified charge-coupled device (iCCD) camera. To accomplish this, a gate mode with a nanosecond exposure is suitable, and the external trigger should be synchronized with the rf power source. In general, the total radiation power during a few-nanosecond period is quite low. Thus, the high gain of a micro-channel plate (MCP) helps increase the signal-to-noise ratio. Additionally, the ‘integrate-on-chip (IOC) mode’ provides the result of the charge accumulation on the CCD during the exposure time, which is useful for obtaining high-quality images. The detailed settings used in these experiments are discussed in the main paper. Care should be taken with the timing generator of an iCCD camera and the voltage probe since instrumental time delays exist. To accurately compare the discharge structures with the voltage waveforms in the phase space, an external trigger was used to compensate for a delay time of 14.7 ns (the signal delay induced by the voltage probe) in the voltage waveforms and 35 ns (the delay induced by the iCCD camera) in the gate signal, as illustrated in Fig. S4(a).

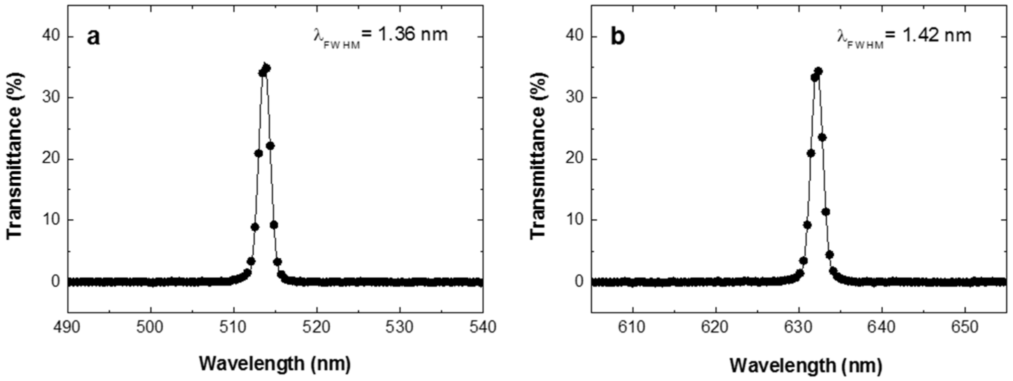

Supplementary Figure S3. Transmittances of the interference filter for the neutral bremsstrahlung measurements. Transmittances of the two filters with center wavelengths of (a) 514.5 nm with a FWHM of 1.36 nm and (b) 632.8 nm with a FWHM of 1.42 nm. The λ_{FWHM} are sufficient to measure the intensity ratio, and the two filters have sufficiently high transmittances (≃ 33%) at the center wavelengths and optical densities (> 4 for both) for other spectral ranges.

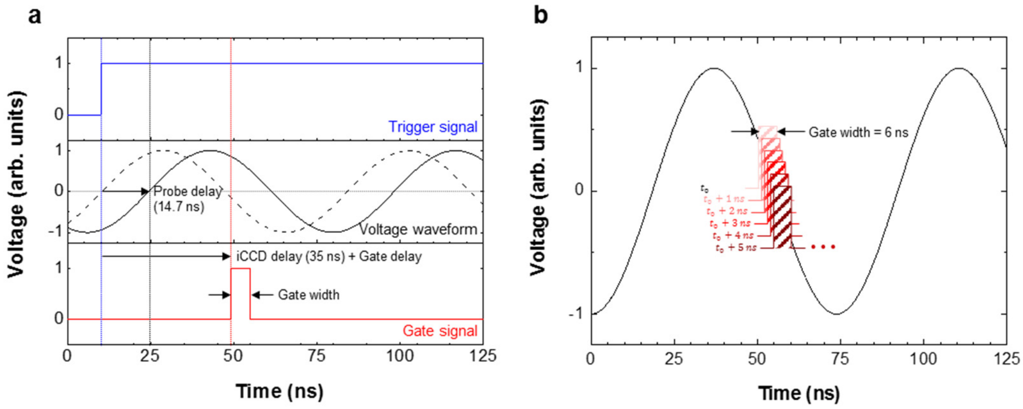

Supplementary Figure S4. Signals for the iCCD camera measurement. (a) Timing chart of a trigger signal from a function generator with the discharge voltage measured by a high-voltage probe and the gate signal of an iCCD camera in a time-resolved measurement. The black dash line depicts a voltage waveform compensated for the probe delay. (b) Plot of the sequential gate signals with a 6 ns width and 1 ns delay.

Relevant background - Optical emissions from rf collisional discharges

The selection of the carrier gas, which plays a major role in plasma chemistry and properties, is a primary concern for generating plasma in a laboratory. Argon and helium are the two most widely used gases for atmospheric pressure plasmas. Helium gas has a low breakdown voltage and high diffusivity; therefore, it can produce volumetric plasma more easily than other gases at atmospheric pressure. Because of these reasons and its high ionization potential, helium gas has been commonly used in experiments for low-temperature, atmospheric-pressure plasmas. In addition to helium, argon is also more attractive than other gases and has historically been one of the most widely used working gases in both low-pressure and atmospheric-pressure plasmas.[3,4]

The emission spectra of helium and argon rf capacitive discharges with 160 W and 380 W input powers, respectively, were analysed, and each intensity peak was identified, as detailed in Fig. S5. The input power is not important in this quantitative analysis. Both spectra clearly show continuum radiation over the entire spectral range. Although the plasmas were produced inside an airtight, stainless-steel chamber, either a small amount of air and water vapour diffused into the discharge area or residual air impurities inside the chamber affected the plasma. Consequently, numerous molecules and atoms other than helium or argon were detected in the spectra. The possible sources of the water molecules were the chamber wall and impure supplying gas. The hydrogen Balmer-α and Balmer-β lines, whose line broadening provides information on n_e, were only observed in the helium discharge. In addition to the hydrogen lines, a substantial number of atomic lines and molecular bands were observed in the helium discharge, whereas only a few radiators were noticeable in the argon discharge spectrum. Metastable atoms play a decisive role in defining the physical and chemical properties of plasma, especially in atmospheric-pressure plasmas, due to their high concentrations. Thus, metastable atoms can be a major contributor to the differences observed between the spectra. In high collision plasmas, metastable atoms are rapidly quenched by heavy molecules through frequent collisions; as a result, hydrogen atoms are actively produced from the dissociation of water molecules via reactions with metastable atoms. The three main quenching reactions of the metastable atoms (A^*), where A is helium, argon, or another carrier gas, by water molecules are as follows,[5]

$$A^* + \mathrm{H_2O} \rightarrow A + \mathrm{H} + \mathrm{OH}\ \left(\mathrm{X}^2\Pi \,\mathrm{or}\, \mathrm{A}^2\Sigma^+\right), \qquad \text{(S1)}$$

$$A^* + \mathrm{H_2O} \rightarrow A + \mathrm{H_2} + \mathrm{O}, \qquad \text{(S2)}$$

$$A^* + \mathrm{H_2O} \rightarrow A + 2\mathrm{H} + \mathrm{O}. \qquad \text{(S3)}$$

The branching ratios of the above reactions (S1-S3) with Ar^* are 0.54±0.04, 0, 0.46±0.05, respectively, and those for the formation of OH($\mathrm{X}^2\Pi$) and OH($\mathrm{A}^2\Sigma^+$) via reaction (S1) are 0.26±0.09 and 0.28±0.09, respectively. Thus, the presence of hydrogen atoms in the plasma can be indirectly confirmed by the large OH peak at 308.9 nm ($\mathrm{A}^2\Sigma^+ - \mathrm{X}^2\Pi$). The hydrogen atom yields of metastable argon, krypton, xenon are 1.52±0.1, 1.08±0.06, 1.00±0.06, respectively; a higher-energy metastable atom produces more hydrogen atoms. From this information, although the hydrogen atom yields of He^* were not reported[5], He^* is estimated to produce more hydrogen atoms in plasma than Ar^*. Furthermore, because the lowest energy level of He^* (19.8 eV) is much higher than that of Ar^* (11.55 eV), electronically excited hydrogen atoms and other species are easily created in helium plasmas. Therefore, spontaneous emissions from air impurities and related substances are readily observed in helium discharges. It is worth mentioning that the argon capacitive discharge is favoured for n_e and T_e measurements using neutral bremsstrahlung-based electron diagnostics because a wide, line-free spectral region is observed in the argon discharge; in contrast, atomic lines and molecular bands are densely distributed over the entire wavelength range in the helium discharge. Moreover, as described below, the plasma imaging technique for the 2-D distribution of T_e is more easily applied in argon plasmas.

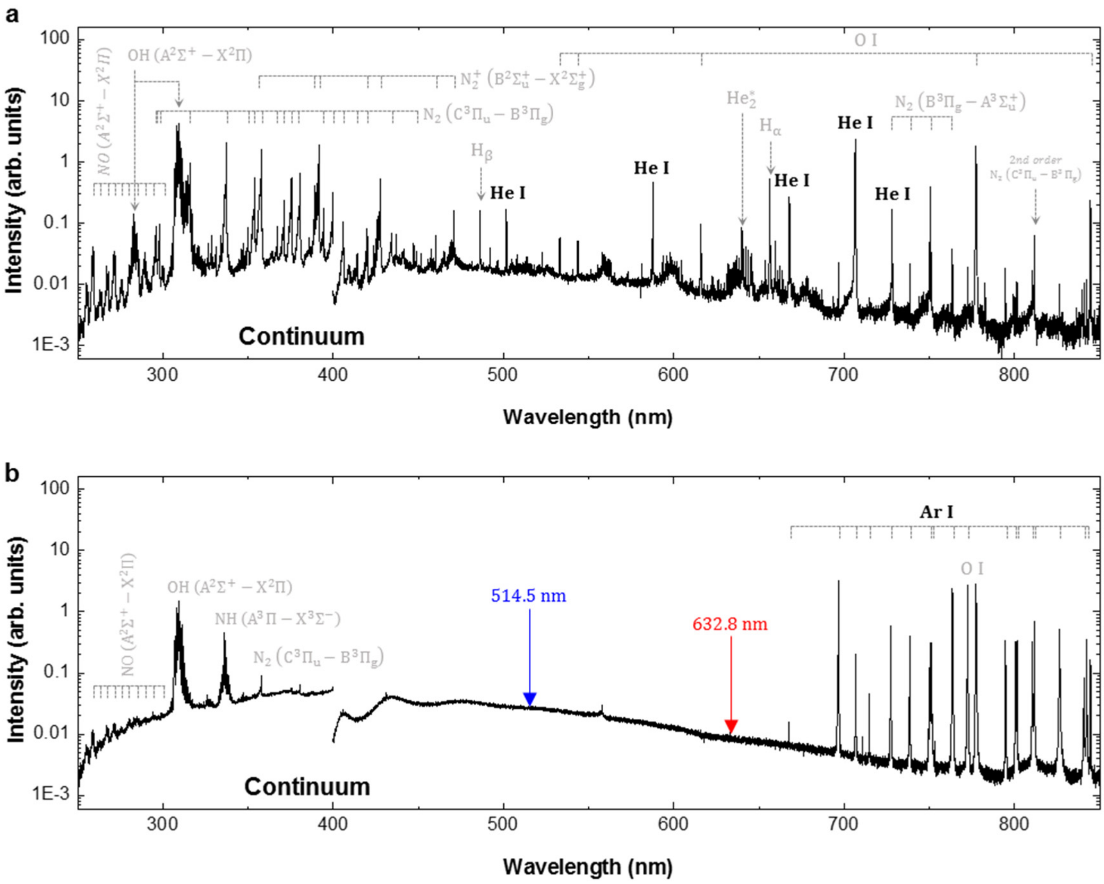

Supplementary Figure S5. Results of the optical emission spectroscopy of rf capacitive discharges at atmospheric pressure. Raw optical emission spectra of (a) helium and (b) argon rf capacitive discharges in the spectral range of 250 – 850 nm. For these spectra, the plasma emission near the electrode (sheath edge), where the intensity of the atomic lines is the local maximum, was corrected and measured using a SpectraPro-750 monochromator with a slit width of 10 μm. A discontinuity exists at 400 nm because the spectra in the wavelength range 400 – 850 nm were measured separately using a 400-nm long-pass filter to eliminate the second-order emission from 200 – 400 nm.

Supplementary References

1. Raizer, Y. P., Shneider, M. N. & Yatsenko, N. A. Radio-Frequency Capacitive Discharges (Newyork: CRC, 1995).
2. Park, S., Choe, W., Kim, H. & Park, J. Y. Continuum emission-based electron diagnostics for atmospheric pressure plasmas and characteristics of nanosecond-pulsed argon plasma jets. *Plasma Sources Sci. Technol.* **24**, 034003 (2015).
3. Moon, S. Y., Choe, W. & Kang, B. K. A uniform glow discharge plasma source at atmospheric pressure. *Appl. Phys. Lett.* **84**, 188-190 (2004).
4. Godyak, V. A., Piejak, R. B. & Alexandrovich, B. M. Electrical characteristics of parallel-plate RF discharges in argon. *IEEE Trans. Plasma Sci.* **19**, 660-676 (1991).
5. Balamuta, J. & Golde, M. F. Quenching of metastable Ar, Kr, and Xe atoms by oxygen-containing compounds: A resonance fluorescence study of reaction products. *J. Chem. Phys.* **76**, 2430-2440 (1982).
